# Nervous Diseases

**Published:** 1893-10

**Authors:** James Wright Putnam

**Affiliations:** Clinical Professor of Nervous Diseases, Medical Department, University of Buffalo


### NERVOUS DISEASES.

Conducted by JAMES WRIGHT PUTNAM, M. D.,

Clinical Professor of Nervous Diseases, Medical Department, University of Buffalo.

### SPASMODIC TORTICOLLIS—TREATMENT BY EXCISION OF NERVES.

NOBLE SMITH (*The Lancet*, August 26, 1893). In this article five cases are reported. In Case I., after four years, there is no pain in the neck; there have been no spasms since the operation. The spine has become straight. Motion of head is good, sensation is normal, and the appearance of the neck is natural. This is the result in a case of sixteen years' duration, in whom the spinal accessory of the left side was operated on by excision of a piece. Later, the same was done on the right side to the posterior branches of the second, third, and fourth cervical nerves.

CASE II.—A man, fifty-seven years old, had, in addition to spasm of muscles on both sides of the neck, spasm of muscles of the face. Two years after operation he wrote: "I am cured of the distressing malady on the side of the head you operated upon, but the other side gives me great trouble."

CASE III.—Patient aged forty-five; duration of disease, fourteen years; was free of pain one and one-half years after operation.

CASE IV.—A woman, past sixty years, had spasms over fourteen years. Excision of the left spinal accessory, and of the posterior cervical nerves on both sides, resulted in perfect freedom from spasms, although the strength of the neck has been somewhat impaired.

CASE V.—Showed improvement after operation.

These results certainly show that excision of the nerve, for this obstinate malady, is justifiable in obstinate cases.

---

### FOR MYXEDEMA.

THE *Alienist and Neurologist*, July, 1893. (Abstracts from *The Lancet*, February 4, 1893.) The following are the methods of employing the thyroid juice in this affection: One-eighth of a thyroid gland, in powder, given two to seven times a week in lukewarm beef tea.—(Arthur Davies). One-half to one thyroid gland daily, eaten raw—(Pasteur). One-half a thyroid gland, three times a week, fried sufficiently to make it palatable—(Calvert). Equal parts of thyroid juice and glycerine, and a five per cent. aq. sol. of carbolic acid. (ʒiss. equal to one sheep's thyroid.) Give m. x.–xv. hypodermically, or ʒi. by mouth—(Murray).

---

### THE MENTAL SYMPTOMS OF MYXEDEMA AND THE EFFECT ON THEM OF THE THYROID TREATMENT.

CLOUSTON (*British Medical Journal*, August 26, 1893). The author reported nine cases of myxedema sent to the Royal Edinburgh Asylum on account of insanity. Two of these had been treated by thyroid extract.

One of the cases had been insane for one year, the other for three years. The myxedema had existed for over five years in both. Both were enfeebled in mental power, this having succeeded exaltation, morbid suspicion, and excitement at the beginning of the attacks of insanity. One case was cured in four months, the other in six. A slow intermittent treatment, by small doses (one-sixteenth of a thyroid twice a week), was found to be the best. In both cases the mental symptoms disappeared with the bodily symptoms. The whole mental powers acquired strength, and the normal enjoyment of life was restored.

---

### THE SENILE FORM OF MULTIPLE NEURITIS.

OPPENHEIM (*Berlin Med. Wochenschrift*, No. 25). The author gives the result of a study of six cases as follows: The characteristics of the senile form of multiple neuritis are: (1) The absence of the etiological influences, intoxication, and infection; (2) the pronounced chronicity of its course; (3) the absence of the mildness of irritative sensory disturbance; (4) the incompleteness of motor and sensory impairment; (5) the freedom from involvement of the cerebral nerves.

As a result of treatment, two cases were almost completely restored, a third was decidedly improved, the fourth was made worse. No change was observed in the other two.

---

### OBSTETRIC PARALYSIS.

CARTER (*Boston Medical and Surgical Journal*, May 4, 1893). Thirty-two cases are tabulated by the author, including sixteen of his own. He concludes that paralysis is due to a stretching of the nerve trunk, formed by the fifth and sixth cervical nerves, by traction on the head or pressure on the breach when the head is retarded, or by traction on the shoulder when the head is retarded. It is not caused by pressure from the forceps. The paralyzed muscles are the deltoid, supraspinatus, infraspinatus, teres minor, biceps, and brachialis anticus with the supinators. In some extreme cases the paralysis includes the extensors of the wrist and fingers.

The arm is rotated internally, the elbow points outward. The paralysis is not noticed before the second or third day. Reaction of degeneration is present after a few days. Treatment consists of passive movements, massage, galvanism every other day through the affected muscles, and brachial plexus.

The prognosis is good, though recovery is sometimes delayed for months and even years. Permanent disability is very rare.

---

### MENIERE'S DISEASE, CURED BY SALICYLATE OF SODIUM.

GAY (*British Medical Journal*, September 9, 1893). The writer reports the case of a lady, aged 65, who had suffered since 1890· Was treated with gelsemium without much benefit. The patient was put on three-grain doses of soda salicylate, the fits being at once reduced in frequency, but continued for some months. March, 1891, saw her much better, but she suffered from heart weakness in May, 1891. In June she had one fit. In November, 1891, she considered herself cured, and has since remained free except slight deafness in one ear.

Treatment lasted rather more than a year, salicylate being taken daily. During the last six months, however, she had considerable intervals without it.

EFFECT OF OPIUM HABIT IN THE MOTHER ON THE NEW-BORN.

Dr. Wood (*Journal of Inebriety*, July, 1893,) says: "When a mother uses opium, the child in the womb has the opium habit at the time of birth." It has frequently been noted that when children are born in women who are opium eaters, the child seems at first well; then, in the course of a few hours, goes into a collapse and dies. Now, I believe that the collapse in these cases develops because the child has not the usual stimulus of opium, and should the child be given opium immediately after birth, it would live.

